# A Present Day Approach to Crown Lengthening – Piezosurgery

**DOI:** 10.7759/cureus.6241

**Published:** 2019-11-26

**Authors:** Vamsi Lavu, Chakravarthy Arumugam, Nivedha Venkatesan, Balaji SK, Giri Valandhan Vedha

**Affiliations:** 1 Periodontology, Sri Ramachandra Institute of Higher Education and Research, Chennai, IND; 2 Conservative and Endodontics, Sri Ramachandra Institute of Higher Education and Research, Chennai, IND; 3 Periodontics, Sri Ramachandra Institute of Higher Education and Research, Chennai, IND; 4 Oral Surgery, Sri Ramachandra Institute of Higher Education and Research, Chennai, IND

**Keywords:** crown lengthening, biological width, osseous reduction, piezosurgery, width of attached gingiva

## Abstract

Maintaining a healthy periodontium during restorative procedures is an indispensable condition for achieving optimal functioning and esthetics. Thus, the knowledge of anatomy, influence of the restorative material and its complement on periodontium is vital. Difficulty in maintaining adequate biological width (BW) is a frequent problem encountered in this type of reconstruction. Crown lengthening is commonly used to maintain the dentogingival complex in optimal conditions and to correct aesthetic defects through a smile design. Piezosurgery, which uses a modulated ultrasonic frequency, permits highly precise and safe cutting of hard tissue. Because of its highly selective and accurate nature, its use may be extended to more complex surgical and interdisciplinary cases. In this case report, we present the contemporary use of piezosurgery for crown lengthening procedure.

## Introduction

Crown lengthening is a surgical procedure devised to increase the extent of supragingival tooth structure for restorative or esthetic purposes or a combination of both, by apically positioning the gingival margin, removing supporting bone, or both. The concept of tooth lengthening was first introduced by D. W. Cohen in 1962 [1]. The procedure is based on the principle of maintenance of adequate keratinized gingiva (KG) around the tooth and establishment of biologic width (BW).

Gargiulo et al. in 1961, defined biologic width as the dimension of soft tissue that is attached to the portion of the tooth coronal to the alveolar bone crest [2]. Vacek et al. in 1994, suggested that BW increases anterioposteriorly (1.75 mm-2.08 mm) [3]. The width of keratinised gingiva is also pivotal in maintaining a healthy periodontal status. An adequate width of KG should be maintained around a tooth (≥2 mm) for gingival health whenever possible.

Factors that need to be taken into consideration for undertaking crown lengthening include excessive gingival display (EGD), altered passive eruption (wherein the alveolar crest is ≤2 mm from the cementoenamel junction [CEJ]), lack of tooth structure height, or access for restorative purposes [4]. Crown lengthening is thus indicated in conditions involving teeth with caries extending subgingivally or with extensive carious involvement, tooth fractures, and short clinical crowns caused by incomplete exposure of the anatomic crowns [5].

The literature is replete with several techniques that have been used to achieve the objective of crown lengthening. These include soft tissue gingivectomy, crown exposure by a combination of soft and hard tissue resection and orthodontic extrusion of the tooth. A contemporary approach for soft and hard tissue removal involves use of lasers and piezosurgery. Piezosurgery comprises of directed electrically generated vibrations, providing precise cutting of hard tissues at a controlled rate with no/minimal soft tissue damage. This case report discusses the procedure of crown lengthening done with piezosurgery equipment in a difficult to access area of the oral cavity.

## Case presentation

A 30-year-old male patient was referred to the Department of Periodontics & Implantology, Sri Ramachandra Institute of Higher Education & Research for management of inadequate crown height in relation to tooth no. 27. His past medical history was not contributory; he had undergone root canal treatment one year back at a private dental clinic and came with a complaint of dislodged crown. On intraoral examination, endodontically treated 27 with fractured palatal cusp was evident. A comprehensive periodontal examination was done.

A clinical examination was performed and the parameters are summarized in Table 1.

**Table 1:** Pre-operative clinical parameters for upper left second molar CH: Crown height; PD: Probing depth; WKG: Width of keratinised gingiva; WAG: Width of attached gingiva; TGP: Transgingival probing; BW: Biological width; NA: Not applicable; MB: Mesiobuccal; MIB: Midbuccal; DB: Distobuccal; MP: Mesiopalatal; MIP: Midpalatal; DP: Distopalatal.

Tooth no: 27	MB	MIB	DB	MP	MIP	DP
CH	3 mm	4 mm	3 mm	2 mm	1 mm	0 mm
PD	1 mm	1 mm	1 mm	1 mm	1 mm	1 mm
WKG	3 mm	3 mm	3 mm	NA	NA	NA
WAG	2 mm	2 mm	2 mm	NA	NA	NA
TGP	3 mm	3 mm	2 mm	2 mm	2 mm	2 mm
BW	2 mm	2 mm	1 mm	1 mm	1 mm	1 mm

**Figure 1:**
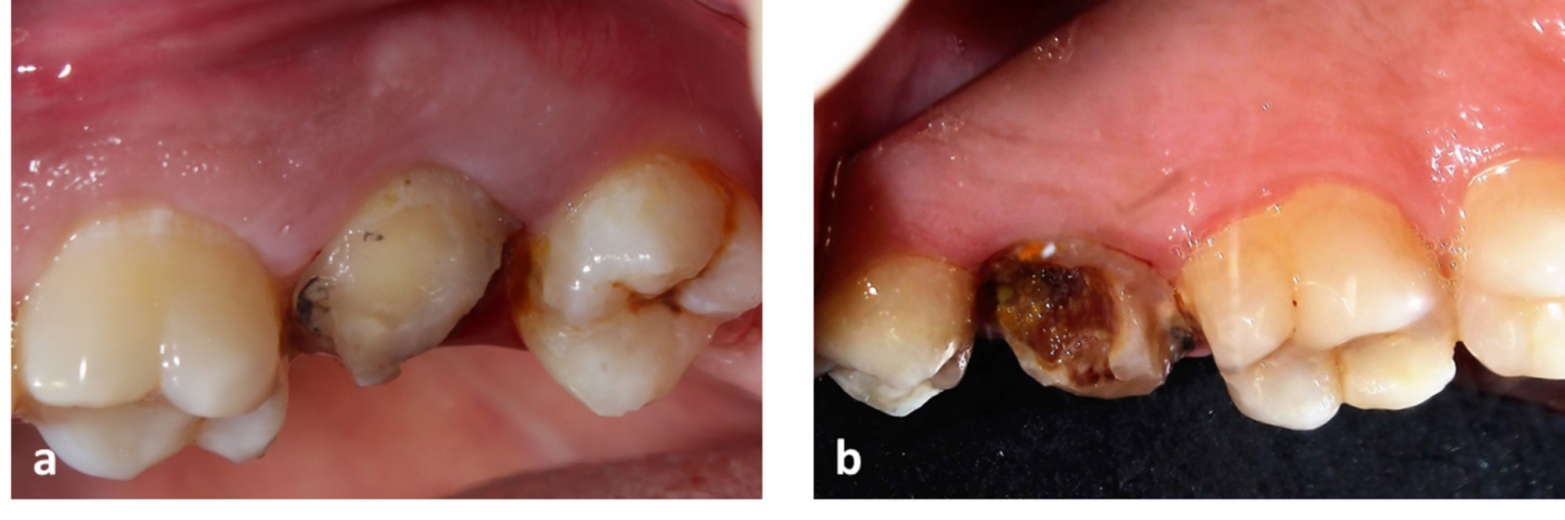
Pre-operative view of the upper left second molar (a) Shows the buccal view of the upper left second molar. (b) Shows palatal view of upper left second molar.

Clinical crown height on the buccal aspect was 4 mm and on palatal aspect was 1 mm (Figure 1).

Probing depth was 1 mm, biological width measured by trans-gingival probing under local anaesthesia was found to be inadequate (<2 mm) in the distal and palatal aspects of tooth. Hence flap procedure with osseous reduction by piezosurgery was preferred.

Surgical technique

**Figure 2:**
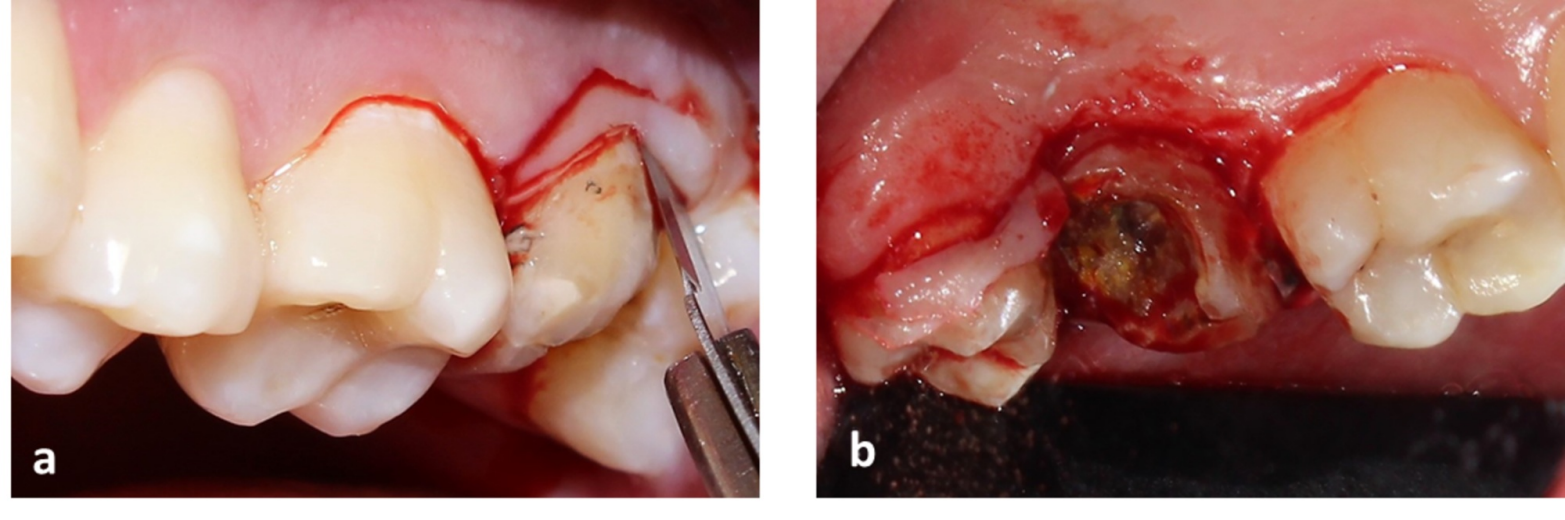
Internal bevel incision placed in relation to upper left second molar (a) Shows incision placed along the buccal aspect. (b) Shows incision placed along the palatal aspect of upper left second molar.

Under local anesthesia (2% Lidocaine with adrenaline 1:200000 concentration), internal bevel incision of 2 mm buccal and 3 mm in the palatal aspect was given (Figure 2).

**Figure 3:**
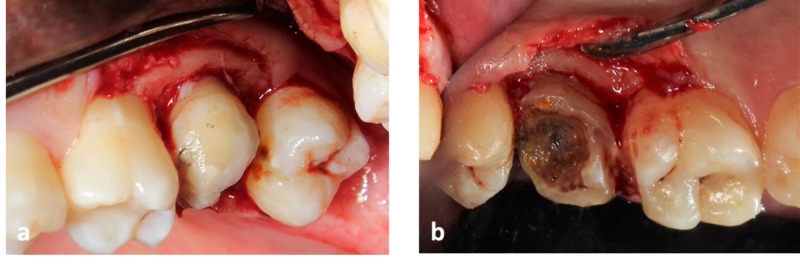
Post-operative view of upper left second molar after osseous re-contouring (a) Shows buccal view. (b) Shows palatal view of upper left second molar.

**Figure 4:**
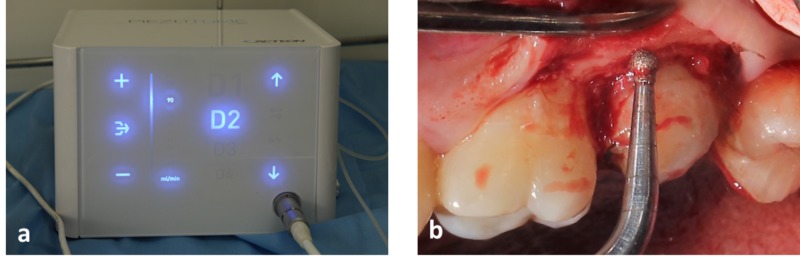
Osseous reduction done with Piezotome CUBE (a) Shows Piezotome CUBE setting used. (b) Shows the use of Piezotome tip CE2 in relation to upper left second molar.

This was followed by crevicular incision and elevation of a full thickness periosteal flap. The marginal cuff of tissue was removed using an area specific Gracey curette (#11-12). Osseous reduction (2 mm) was carried out to increase the crown height and to establish adequate biological width. Piezosurgical tips CE1 (ball diameter - 1.75 mm) and CE2 (ball diameter - 1.20 mm) were used to recontour the bone around the tooth with light sweeping strokes. D2 setting was used for the procedure with a controlled flow of sterile saline (Flow rate - 80 ml/min) in the Piezotome (CUBE®) equipment (Figures 3, 4).

**Figure 5:**
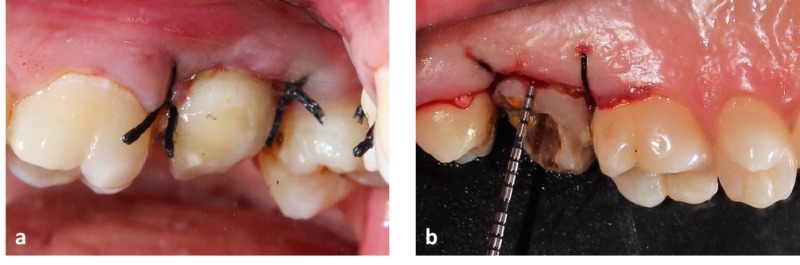
Simple interrupted sutures placed in relation to upper left second molar (a) Shows simple interrupted sutures placed, buccal view. (b) Shows palatal view of the upper left second molar.

Flaps were then approximated with 3-0 BBS sutures (Figure 5).

**Figure 6:**
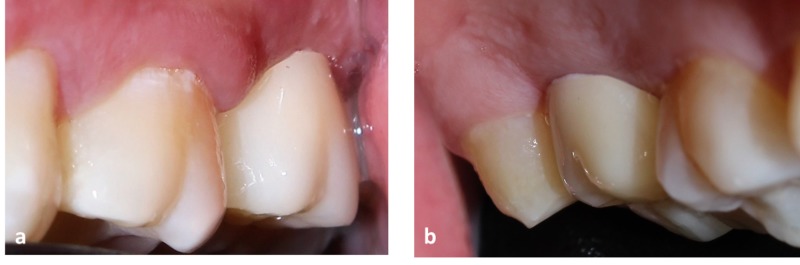
Post-operative view of upper left second molar with the final ceramometal crown in place (a) Shows the post-operative buccal view. (b) Shows post-operative palatal view in relation to upper left second molar.

Post-operative medications prescribed comprised of antibiotic cap. Amoxcillin 500 mg, thrice daily and analgesic combination of T. Aceclofenac+Paracetamol, twice daily for three days following the surgery. Twice daily use of Chlorhexidine oral rinse 0.2% was recommended for two weeks, along with other pertinent postoperative instructions. The postoperative healing was uneventful and the sutures were removed on the 10th postoperative day. Two weeks post-surgery, the tooth preparation was modified to receive for a full coverage ceramometal crown with a laser sintered coping. The fabricated crown was cemented with adhesive polyalkenoate cement after verifying the marginal fit and occlusal harmony (Figure 6).

## Discussion

Piezoelectric surgery was first made known by French Jacques and Pierre Curie in 1880 [6]. Since then ultrasonics have been widely known as a treatment modality for cutting bone. Piezoelectric effect refers to a phenomenon wherein electrical tension on crystal and ceramic materials such as quartz results in oscillations of ultrasonic frequency [7]. The vibrations produced are amplified and transferred to a vibrating tip which when applied on bone tissue with slight pressure results in cavitation phenomena. This cutting effect is observed exclusively in mineralized tissue and the procedures which utilized his effect were termed Piezosurgical procedures.

Compared with traditional rotary instrumentation, piezosurgery requires less hand pressure allowing for enhanced operator sensitivity and control. Microvibrations produced by piezosurgery are low frequency modulated vibrations at 25 to 30 khz, which selectively cut the bone without damaging adjacent soft tissues [8]. Owing to limited vibration amplitude (max. 200 μm) and the design of osteotome tips for specific surgical situation, it offers precise micrometric bone cuts [9]. The cutting action is less invasive, producing minimal collateral tissue damage, which results in better healing. Owing to its cavitation effect on physiological solutions, piezosurgery creates a virtually bloodless surgical site that makes visibility in the working area much clearer than with conventional bone cutting instruments [10].

Localized bone necrosis has been reported when the temperature exceeds 47 degrees Celsius for one minute due to the contact of rotating tools [11]. Harder et al. observed that the critical temperature rises only when the irrigation volume is as low as 20 ml per minute [12]. Unlike conventional burs and micro saws, piezosurgery inserts with the help of internal irrigation reduces the risk of postoperative necrosis [13].

Piezosurgery not only cuts the hard tissue selectively, but also produces a haemostatic effect on the surrounding tissue. Thus, it can be put to use in sites where bone is in close proximity to vital and delicate structures such as nerves, blood vessels or the sinus mucosa [14].

Crown lengthening in this present case report was done for the upper left second molar tooth which represents an area of limited access. Since the risk of soft tissue damage is minimal with piezosurgery tips, this method was preferred for carrying out the procedure. The patient healing was uneventful with minimal post-operative discomfort with no report of swelling of gums/post-operative bleeding.

Previous microtopographic and histomorphometric studies have shown that the bone surface cut using piezoelectric tips showed no sign of adverse reactions in the mineralized tissues and live osteocytes were observed with no sign of cellular affliction [15]. Piezosurgery also seems to be more efficient in the first phases of bony healing. A histomorphological study revealed that the piezosurgery increased the concentration of bone morphogenic protein (BMP-4), TGF beta-2, tumor necrosis factor and interleukin-1, 10 and decreased some of the pro-inflammatory cytokines in the bone while stimulating bone remodelling as early as 56 days after treatment [16]. Thus, neo-osteogenesis was shown to be consistently more active in cases where piezosurgery is used [17].

The crown lengthening technique performed with piezosurgery using appropriate inserts makes it possible to effectively reduce bone while preserving root surface integrity. The rate of postoperative wound healing (baseline and 14, 28, and 56 days after surgery) in a dog model following surgical ostectomy and osteoplasty has been used to gauge the efficacy of the piezosurgery instrument compared with a rotary instrument, i.e., carbide bur or a diamond bur. The osseous repair and remodelling following periodontal resective therapy with piezosurgery was found to be more favorable than pruning with carbide or diamond burs [18].

In a study by Romeo et al., four devices - Erbium: Yttrium Aluminium Garnet [Er: YAG] laser (2.94 mm), piezosurgery, high‑speed drill and low‑speed drill - were compared for the peripheral bone damage induced by them. Four different parameters were analyzed: cut precision, depth of incision, peripheral carbonization and presence of bone fragments. Er:YAG sections showed no significant peripheral carbonization. The edges of the incisions were always well‑shaped and regular, no melting was observed. The piezosurgery specimens had superficial incisions without thermal damage but with irregular edges. The sections obtained by traditional drilling showed poor peripheral carbonization especially if cut at a lower speed [19].

The quantitative roughness analysis of osteotomized bone surfaces prepared by conventional osteotomy and piezoelectric technique was assessed. It was concluded that the ultrasonic technique preserved the original bone structure and the superficial roughness was minimum for the piezo‑osteotomy surface followed by the microsaw and Lindemann bur osteotomy surfaces [20].

## Conclusions

This case report highlights the application of contemporary technology of piezosurgery for the crown lengthening done by flap with ostectomy. Crown-lengthening is a very viable option for facilitating restorative therapy or in improving esthetic appearance. When planning a crown lengthening procedure, the dentist should evaluate the patient’s complete periodontal condition and disclose all possible treatment options to the patient and piezosurgery has been proven to have a potential role in osseous surgery with minimal post-operative complications and maximum patient comfort.
